# CDSbank: taxonomy-aware extraction, selection, renaming and formatting of protein-coding DNA or amino acid sequences

**DOI:** 10.1186/1471-2105-15-61

**Published:** 2014-02-28

**Authors:** Bart Hazes

**Affiliations:** 1 Department of Medical Microbiology & Immunology, 6-020 Katz Group Centre, University of Alberta, Edmonton, Alberta T6G 2E1, Canada

**Keywords:** Web server, Genbank, Redundancy control, Quality control, Taxonomy

## Abstract

**Background:**

Protein-coding DNA sequences and their corresponding amino acid sequences are routinely used to study relationships between sequence, structure, function, and evolution. The rapidly growing size of sequence databases increases the power of such comparative analyses but it makes it more challenging to prepare high quality sequence data sets with control over redundancy, quality, completeness, formatting, and labeling. Software tools for some individual steps in this process exist but manual intervention remains a common and time consuming necessity.

**Description:**

CDSbank is a database that stores both the protein-coding DNA sequence (CDS) and amino acid sequence for each protein annotated in Genbank. CDSbank also stores Genbank feature annotation, a flag to indicate incomplete 5′ and 3′ ends, full taxonomic data, and a heuristic to rank the scientific interest of each species. This rich information allows fully automated data set preparation with a level of sophistication that aims to meet or exceed manual processing. Defaults ensure ease of use for typical scenarios while allowing great flexibility when needed. Access is via a free web server at http://hazeslab.med.ualberta.ca/CDSbank/.

**Conclusions:**

CDSbank presents a user-friendly web server to download, filter, format, and name large sequence data sets. Common usage scenarios can be accessed via pre-programmed default choices, while optional sections give full control over the processing pipeline. Particular strengths are: extract protein-coding DNA sequences just as easily as amino acid sequences, full access to taxonomy for labeling and filtering, awareness of incomplete sequences, and the ability to take one protein sequence and extract all synonymous CDS or identical protein sequences in other species. Finally, CDSbank can also create labeled property files to, for instance, annotate or re-label phylogenetic trees.

## Background

Protein sequences are frequently used for molecular phylogeny and protein structure-function studies. The rapid growth of sequence databases increases the power of these methods but the preparation of large sequence data sets becomes a non-trivial task. In a typical workflow, protein sequences are selected either by homology to a query of interest or by a search for specific sequence annotation or features. Amino acid sequences can be readily retrieved because each is associated with a unique identifier. However, accessing the corresponding DNA sequence is not straightforward because DNA sequence identifiers do not map to individual CDS but to the typically longer nucleotide sequence that contains the CDS. Protogene can extract CDS but is slow or requires users to prepare template files [1]. The EMBL maintains a CDS database [2], but dbfetch (https://www.ebi.ac.uk/Tools/dbfetch/) limits downloads to 200 sequences, does not cover the popular RefSeq database [3] and does not interface well with results from the NCBI blast-family of programs.

A typical second step in the workflow reduces redundancy by representing clusters of closely related sequences by a single representative. Existing methods cluster sequences at a user-defined sequence identity threshold [4] but normally give little control over the sequence chosen to represent each cluster. Here an expert user would take sequence completeness, quality, length, and preferred species into account to select the best representative for each cluster. However, for large data sets this becomes intractable without automation. CDSbank exploits its stored knowledge to mimic and automate an expert user’s decision making process. CDSbank also implements an alternative clustering method based on taxonomy. This is particularly useful for phylogenetic studies where the user can request one representative per species, genus, family, etc.

The opposite problem, too little redundancy, arises when proteins with identical amino acid sequence exist in different species or are encoded by distinct but synonymous CDS. This diversity is lost in non-redundant protein databases, such as the NCBI NR database, but is important for taxonomic studies or analyses of CDS such as positive selection. To my knowledge there are no tools to easily recover lost taxonomic or CDS diversity and this was one of the design goals of CDSbank.

The final step in preparing sequence data is to give each sequence an informative label, often indicating taxonomy and/or gene name. The REFGEN server [5] can generate sequence labels based on accession code and/or species name, but only if this information is present on the original fasta header line. Moreover, REFGEN assumes that names follow the binomial species nomenclature, which fails for many hybrids, viruses, or subspecies names such as *Homo sapiens neanderthalensis*. CDSbank instead has access to the original Genbank annotations and is fully taxonomy aware.

The CDSbank webserver was designed to address the many practical problems in a manner that meets and sometimes exceeds the performance of an expert user, while remaining easy to use. It has been tested extensively by students in a bioinformatics course. For typical applications, the user simply indicates which sequences to retrieve and select a predefined label plus sequence representation. If needed, optional sections on the web form give full control over sequence selection and formatting.

## Construction

### Construction and content

The main CDSbank database is built by parsing all Genbank nucleotide sequence data files from the BCT, CON, INV, MAM, PHG, PLN, PRI, ROD, VRT, VRL divisions, as well as the reference sequence (RefSeq) and whole genome shotgun (wgs) databases [6]. Amino acid sequences without corresponding DNA sequence, including protein data bank entries, non-natural sequences, and Genbank divisions without CDS annotations are not included (EST, GSS, PAT, SYN, TSA, UNA). During processing, CDSbank extracts information from the primary source feature (Additional file 1: Table S1) and each CDS feature (Additional file 1: Table S2), except pseudo genes and CDS without translation. CDSbank also generates extra annotation (Additional file 1: Table S2), for instance a flag to indicate if the 5′ end or 3′ end, or both, are incomplete based on the feature location specification. This allows for safe automatic rejection of partial sequences.

All entries are indexed based on both their protein accession code and GI code. In addition, MD5 hash values for the amino acid and DNA sequence are used as compound indices with the NCBI taxonomy ID. This allows efficient retrieval of entries based on sequence identity, with or without restriction to a specific taxon.

CDSbank builds a separate database with the full NCBI taxonomy. Taxon records are indexed by the taxonomy identifier and store both common and scientific names. Each entry represents a node in the taxonomy tree while pointers to parent and child nodes permit tree traversal, for instance to construct an entire taxonomic lineage. In addition, as it parses each annotated CDS, CDSbank increments a counter for the corresponding taxon record in the taxonomy database. The number of CDS for a taxon is used as a proxy to rank its relative scientific interest.

All data is stored with mongoDB (http://www.mongodb.org/), a highly efficient non-relational database that stores records as indexed dictionaries. CDSbank is rebuild fully after each Genbank release followed by daily updates. It currently contains over 88 million CDS entries (Genbank release 200 updated to 27/2/2014).

### Utility

#### Sequence selection

CDSbank can retrieve sequences and annotation based on unique identifiers, one per line, or a set of sequences in fasta format. The NCBI accession code is the preferred sequence identifier because it remains valid even if a sequence is updated. The NCBI GI code can also be used but becomes obsolete after a sequence entry is updated because CDSbank only stores the latest version. Identifiers from other databases are not supported but users can still gain access to CDS sequences and annotation by simply uploading their sequences, DNA or protein, in fasta format. CDSbank will use the MD5 hash value of the sequence as a generic index and retrieve all entries that share the exact same sequence. Moreover, if the fasta title line lists the species name in square brackets then CDSbank will restrict the database matches to that species. Sequence lookup is case insensitive but has to be otherwise exact. Finally, if no sequence match is found, CDSbank will use the sequence as provided but without access to annotation, except taxonomy if the species name is listed in square brackets on the title line. The recommended and most robust method is to upload fasta-formatted sequences with a NCBI-generated title line. CDSbank will first use unique identifiers on the title line but can still fall-back to using the sequence and species name if needed.

The user can specify the set of desired sequences by either uploading a file or entry into a text window. Specification of the sequence selection is the only mandatory user input; all other sections discussed below are optional.

#### Sequence expansion

CDSbank can use the MD5 hash of the protein sequence as an index to find all entries that share the identical sequence. The expanded set represents the full taxonomic coverage and all synonymous CDS variants. The user can also request expansion of CDS variants without expanding taxonomic coverage. In that case the search uses a mongoDB compound index that combines the MD5 hash and taxonomy identifier. To achieve expansion of just taxonomic diversity, full expansion has to be combined with redundancy removal based on amino acid sequence identity as described in the next section.

#### Redundancy control

CDSbank defines redundancy based on one or more of the following three criteria: accession code, sequence identity, or taxonomy. The default is to use the accession code criterion, which ensures that each accession code is represented only once. Sequence based redundancy removal mimics the NR database where identical entries are represented by a single sequence. A unique feature of CDSbank is that it can also do this on the DNA level to generate a non-redundant CDS sequence set. The latter is useful to suppress redundant CDS sequences generated by synonymous CDS expansion. Sequence-based redundancy control can be combined with taxonomy, such that duplicate sequences in different taxons are retained.

A special feature of CDSbank is that it can also reduce redundancy based on taxonomy itself. A common use is to cluster sequences based on the taxonomy identifier so each taxon is represented by just a single sequence. This mimics the taxonomically non-redundant RefSeq database [3]. As a generalization, one can cluster at any taxonomic level (species, genus, family, superfamily, etc) to ensure that each taxonomic group is represented by a single sequence. This can be particularly useful for phylogenetic studies.

#### Quality control

Sequence databases contain many entries that have been partially sequenced or where the protein and/or CDS sequence contain ambiguous residues. Depending on the application, the user may need to reject all such sequences or, to avoid information loss, reject them only if a superior equivalent sequence is available. CDSbank accomplishes this by integrating quality control with redundancy control. After creating redundant clusters based on the criteria described above, sequences can be flagged as imperfect when: i) their N-terminus is incomplete, ii) their C-terminus is incomplete, iii) their CDS sequence has ambiguous residues, iv) their protein sequence has ambiguous amino acids, and/or v) the sequence length is outside a user-specified range. The default is to flag a sequence if any of the first four problems exist, but this can be fine-tuned by the user.

#### Selecting the optimal cluster representative

If sequence clustering is used and only one sequence passes the quality control tests then that sequence represents the cluster. If multiple sequences pass quality control, CDSbank attempts to find the best representative by selecting, in order, on the following properties until one sequence remains: species importance (using the number of annotated CDS in Genbank to rank species importance), RefSeq membership, number of times the protein sequence is represented in Genbank, CDS sequence length, Genbank source sequence length, order in the user-provided input list. If none of the sequences passed quality control, the default action is to use the sequence with the largest number of non-ambiguous residues as the cluster representative, but the user can indicate to leave such clusters unrepresented.

#### Sequence label and formatting

By default CDSbank produces fasta format DNA sequences but other amino acid or DNA sequence representations, including R/Y wobble base coding, can be selected from a menu. The species common name is the default sequence label, or the scientific name if no common name exists. A menu predefines alternate sequence labels such as: identifiers, species name, gene name, or the original uploaded sequence label. To accommodate idiosyncrasies of downstream processing software, CDSbank can ensure that sequence labels do not exceed a user-defined length and do not contain special characters that may be incompatible with other software. Finally, if identical sequence labels are encountered a number is appended to ensure all labels are unique. These commonly used formatting choices are presented at the top of the web form for easy access.

The label selection menu has one advanced option, “python format”, which gives users direct access to the wealth of information available for each CDS. This is implemented as a python dictionary that associates each piece of information with a unique keyword. Available keywords are listed in Additional file 1: Table S1, S2, and S3. If “python format” is selected the user can access the full power of python formatting strings to define both the sequence label (with controlled length, character set, and uniqueness) and an optional sequence comment. In the python formatting string %(keyword)s statements insert CDS-specific annotation into sequence labels. Users should consult python documentation or CDSbank’s online help page for a full description of python string formatting options. Some common and more creative examples of this flexible mechanism are shown in Table 1.

**Table 1:** Examples of dictionary-based python string formatting

**Label format**	**Comment format**	**Resulting title line**
>%(ComName)s	[%(SciName)s]	>human [Homo sapiens]
>%(genus).1 s%(species)s	gene = %(gene)s	>Hsapiens gene = HBA2
>%(gi)s_%(taxID)s	%(today)s	>450145_9606 2013-05-10
>seq%(sqNr)s	order = %(order)s	>seq1 order = Primates
%(ComName)s	%(pcGC)5.2f	human 65.26^a^

^a^Example of a tab-delimited annotation file linking the sequence label to the GC-percentage of its DNA sequence. In this case, the sequence representation should be set to “do not show sequence”.

#### Labeled property files

For phylogenetic trees it is often desirable to associate simple sequence labels with more informative descriptions or distinct properties of the sequences. After construction of phylogenetic trees, programs such as TREENAMER [5] and iTOL [7] can use this to replace the simple sequence labels with the more informative ones. iTOL can also use numerical and other data to further annotate trees. CDSbank creates labeled property files by setting the sequence representation menu to “do not show sequence” and the sequence label menu to “python format”. As a result a single line is created for each sequence with the sequence label and sequence comment representing label-property pairs. The last row of Table 1 shows how to create GC-content annotation for display by iTOL.

## Discussion

CDSbank was originally developed to efficiently extract large numbers of CDS, which it does. For instance, all 5,431 poxvirus CDS sequences from RefSeq can be downloaded and formatted in just 18 seconds. However, as a bonus of parsing the entire Genbank database, innovative solutions to several other common problems were found. This may be best illustrated by some usage examples.

*Deep phylogeny:* to study distant evolutionary relationships, large numbers of closely related sequences burden the analysis while contributing little information. CDSbank’s taxon-based redundancy reduction can be used to represent taxonomic clades, at any taxonomic rank, by a single sequence. In the process it can reject incomplete or low-quality sequences and select the most-highly sequenced, and thus well-studied, species to represent each clade. This is more objective and efficient than manual selection of clade-representing species and does not require expert taxonomic knowledge. In multi-gene studies, it also avoids the problem that arises when not all genes are sequenced for a clade-representing species. CDSbank will always prefer the gene for the most highly sequenced species, but if a gene is not available for that species the next best representative will be automatically provided as long as at least one member of the clade has the gene sequenced. Finally, to have the sequence label reflect taxonomic clade names, for example at the family rank, use the python format “>%(family)s”, which translates to “>Hominidae” for human sequences. To create a data-provenance table for the methods section in a future manuscript the user runs CDSbank twice. Once to extract the sequences and once more with sequence representation set to “do not show sequence” and python format for label and comment set to “%(family)s” and “%(SciName)s\t%(acv)s”, respectively. This creates a tab-delimited file with columns for family name, species name, and the Genbank protein accession code.

*Positive selection:* positive selection analysis is based on CDS sequences. It is important to include synonymous CDS to avoid underestimating synonymous substitutions. In CDSbank you can upload a unique set of protein sequences and use sequence expansion to extract all CDS that code for each protein. To avoid extracting exact CDS duplicates you can filter redundancy based on the DNA sequence. This will give a complete but non-redundant set of CDS sequences.

*Reliable rejection of partial sequences*: DNA and protein sequences that do not start with a start codon or methionine, respectively, can be recognized as being incomplete. However, sequences can be incomplete even if they have these starting features and there is no easy way to detect sequences that lack part of their 3′ reading frame. The most reliable, but time consuming, solution is to inspect the Genbank feature location definition where “<” and “>” symbols denote incomplete 5′ and 3′ ends, respectively. CDSbank stores this information for each sequence, which allows safe and automated rejection of partial sequences.

## Conclusions

The discussion of special applications above demonstrates the value of flexibility and some of the unique capabilities of CDSbank. However, in most cases CDSbank will be used just as it is intended, a fast and easy to use web server that automates sequence retrieval, curation, and formatting, with minimal user input. This not just saves time and effort, it also stimulates good practice and ensures effective use of the ever growing databases.

## Availability and requirements

The CDSbank server is available free of charge at http://hazeslab.med.ualberta.ca/CDSbank/. For local use, python programs and unix scripts to build and query a custom database can be downloaded from sourceforge. Project name: CDSbank; Project home page: https://sourceforge.net/projects/cdsbank/; Operating system: developed on Linux but without known platform-specific dependencies; programming language Python 2.7; Other requirements: mongodb version 1.8 or higher; Licence: GNU GPL version 3.

## Abbreviations

CDS: Protein coding sequence.

## Competing interests

The authors declare that they have no competing interests.

## Supplementary Material

Additional file 1: Table S1 Annotation collected from the genbank file header and the source feature of the feature table. **Table S2.** Annotation collected, if present, from each CDS feature of the feature table. **Table S3.** Extra sequence annotation. Click here for file
