# Supplementary material for: CDSbank: taxonomy-aware extraction, selection, renaming and formatting of protein-coding DNA or amino acid sequences
Source: BMC Bioinformatics. 2014 Feb 28;15:61. doi: 10.1186/1471-2105-15-61 (PMC3942066; doi:10.1186/1471-2105-15-61)
Supplement: Additional file 1: Table S1 — Annotation collected from the genbank file header and the source feature of the feature table. Table S2. Annotation collected, if present, from each CDS feature of the feature table. Table S3. Extra sequence annotation. [file 1471-2105-15-61-S1.doc]

Table S1: Annotation collected from the genbank file header and the source feature of the feature table.

| **qualifier** | **keyword** | **Comment** |
| --- | --- | --- |
| N.A. ^a^ | gb_date | Last modification date from LOCUS line |
| N.A. ^a^ | gb_length | Nr of bases in the genbank entry from LOCUS line |
| N.A. ^a^ | gb_acc | genbank accession code from VERSION line |
| N.A. ^a^ | gb_gi | genbank gi code from VERSION line |
| mol_type | mol_type | *in vivo* molecule type of sequence |
| chromosome | chr | chromosome number |
| db_xref:taxon | taxID | taxonomy identifier |
| N.A. ^b^ | sub_tax | sample provenance below the taxID level |
| subspecies | sbsp | details on the sample origin of the sequenced DNA below the level of the taxonomy identifier. Listed from more general to more specific. Only qualifiers present in the source feature are stored. |
| strain | strain |
| substrain | sbst |
| cultivar | cult |
| variety | var |
| haplogroup | hapg |
| haplotype | hapt |
| serotype | serot |
| serovar | serov |
| clone | clone |
| subclone | sbcl |
| ecotype | eco |
| pop_variant | popv |
| isolate | isol |
| specimen_voucher | vouch |

*a This information is collected from the genbank header lines and therefore does not correspond to a source feature qualifier.*

^b^ *This keyword is generated by CDSbank based on the subspecies to specimen_voucher qualifiers, taking on the value of the first qualifier that is defined, in the order listed in the table.*

Table S2: Annotation collected, if present, from each CDS feature of the feature table

| **qualifier** | **keyword** | **Comment** |
| --- | --- | --- |
| protein_id | _id ^a^ | NCBI accession code without version number |
| protein_id | v | NCBI accession code version number |
| db_xref:GI | gi | NCBI Gene Identifier code |
| EC_number | EC | Enzyme Commission number |
| codon_start | start | 1, 2 or 3 to indicate reading frame starts at base 1, 2 or 3 |
| gene | gene | gene name |
| locus_tag | locus | name for locus |
| product | prod | name for gene product |
| standard_name | name | standardized name ^b^ |
| translation | AA | amino acid sequence |
| N.A. ^c^ | CDS | protein-coding DNA sequence ^d^ |
| N.A. ^c^ | AH | MD5 hash for the amino acid sequence ^e^ |
| N.A. ^c^ | DH | MD5 hash for the DNA sequence ^e,f^ |
| N.A. ^c^ | loc | feature location definition |
| N.A. ^c^ | NC | flag: 0=complete, 1=5' truncated, 2=3' truncated, 3=5'&3' truncated |

^a^ The keyword _id is use by mongoDB to denote the primary index field

^b^ If this qualifier is absent CDSbank will create it with the content of the gene, locus_tag, or product qualifier (searched for in that order)

^c^ These items are created by CDSbank

^d^ Includes the stop codon

^e^ Hash values are binary and are not available to create sequence labels

^f^ Includes the stop codon

Table S3: Extra sequence annotation a

| **Keyword** | **comment** |
| --- | --- |
| AA | upper case amino acid sequence |
| aa | lower case amino acid sequence |
| AA3 | 3-letter code amino acid sequence |
| DNA | upper case DNA sequence |
| dna | lower case DNA sequence |
| Dna | capitalized codon sequence |
| NNRY | RY-coded codons in upper case |
| pcA, pcG, pcC, pcT | nucleotide composition as percent A, G, C and T, respectively |
| pcAT, pcGC | nucleotide composition as percent A+T and G+C, respectively |
| aa_length | number of amino acids in sequence |
| cds_length | number of nucleotides in sequence (with/without stop codon as requested) |
| seqNr | input order in the user-provided target list |
| fastaTitle | entire fasta header line, without leading “>”, if a sequence was uploaded |
| fastaLabel | first space-delimited word on fasta header line, without leading “>” |
| today | date when CDSbank was run |
| acc | accession code without version number |
| acv | accession code with version number |
| SciName | scientific name |
| ComName | common name |
| taxID | NCBI taxonomy identifier |
| superkingdom | scientific name for the taxonomic rank of superkingdom |
| kingdom | scientific name for the taxonomic rank of kingdom |
| phylum | scientific name for the taxonomic rank of phylum |
| class | scientific name for the taxonomic rank of class |
| order | scientific name for the taxonomic rank of order |
| family | scientific name for the taxonomic rank of family |
| genus | scientific name for the taxonomic rank of genus |
| species | scientific name for the taxonomic rank of species (without genus name) |

^a^ If a keyword with missing value is used for a label it is simply skipped
